# Quantifying How Staining Methods Bias Measurements of Neuron Morphologies

**DOI:** 10.3389/fninf.2019.00036

**Published:** 2019-05-21

**Authors:** Roozbeh Farhoodi, Benjamin James Lansdell, Konrad Paul Kording

**Affiliations:** ^1^Department of Mathematics, Sharif University of Technology, Tehran, Iran; ^2^Department of Bioengineering, University of Pennsylvania, Philadelphia, PA, United States; ^3^Department of Neuroscience, University of Pennsylvania, Philadelphia, PA, United States

**Keywords:** dendritic morphology, staining method, rodent neuroanatomy, neuroinformatics, golgi method, immunostaining, fluorescence microscopy

## Abstract

The process through which neurons are labeled is a key methodological choice in measuring neuron morphology. However, little is known about how this choice may bias measurements. To quantify this bias we compare the extracted morphology of neurons collected from the same rodent species, experimental condition, gender distribution, age distribution, brain region and putative cell type, but obtained with 19 distinct staining methods. We found strong biases on measured features of morphology. These were largest in features related to the coverage of the dendritic tree (e.g., the total dendritic tree length). Understanding measurement biases is crucial for interpreting morphological data.

## 1. Introduction

There are many techniques through which neuron morphologies may be imaged. These techniques can be classified based on two factors: how they target neurons for imaging, and how the axons and dendrites of the probed neurons are labeled so they are visible for imaging. Neurons may be targeted through their propensity to take up heavy metals or genetic markers, through immunohistochemistry or by direct injection (Elston et al., 1999; Jacobs et al., 2001; Travis et al., 2005; Donohue and Ascoli, 2011; Parekh and Ascoli, 2013, 2015; Carter and Shieh, 2015). Neurons may be labeled using a variety of heavy metals, fluorescent or chromogenic labels are used to allow imaging. We may expect that both targeting and labeling aspects will introduce biases upon the resulting reconstructions of morphology.

Staining with heavy metals remains one of the key imaging techniques. Golgi staining is the oldest such method. In Golgi staining, silver nitrate is introduced to fixed tissue, and the metal is taken up by a sub-population of neurons through a mechanism that remains largely uncharacterized. Neurons are stained in their entirety and then imaged with light microscopy (Koyama, 2013). The method can be subdivided into Rapid Golgi, Golgi-Kopsch, and Golgi-Cox (Koyama, 2013) and each version labels a subset of neurons. Other heavy metals such as osmium and lead can instead be used for dense labeling, which is popular for EM data (Watson, 1958; Tapia et al., 2012). Or, alternatively, lipophilic dyes such as DiOlistics can be introduced ballistically to neurons and allow for Golgi-like staining (Staffend and Meisel, 2011). Since the biological mechanism of heavy staining method is mainly unknown, the extract morphology may be subject to selection bias (Staffend and Meisel, 2011). Because it is relatively simple to perform compared to other methods, heavy metals are a popular staining method for fixed tissues.

A more recent approach is to target neurons through genetic markers. Fluorescent proteins such as green fluorescent protein (GFP), red fluorescent protein (RFP), and yellow fluorescent protein (YFP) can be introduced transgenically to be expressed in neurons, and then imaged through fluorescent microscopy to reveal morphology (Marshall et al., 1995). The use of fluorescent proteins may be limited to animals for which good genetic tools exist. Fluorescent proteins are introduced under the control of promoter regions that are active for known cell-type markers. They target specific populations of neurons, which may differ from those neurons targeted by other methods. The size of these potential selection biases again remains relatively uncharacterized. Fluorescent techniques are popular because they readily integrate both into genetic and physiological approaches.

Immunostaining has advanced to be a leading staining technique. Immunostaining uses antibodies to target neuronal molecular markers, which can be labeled with fluorescent or chromogenic tags for imaging (Chen et al., 2010; Tanapat, 2013). A common approach relies on biotin variants, such as biocytin or neurobiotin, being conjugated to an antibody (Swietek et al., 2016). A complex of biocytin and its binding partner, avidin, are tagged with a fluorescent or colored label that can then be imaged. The avidin-biotin complex allows imaging through light, fluorescent, or EM microscopy depending on the label. Common fluorescent dyes used with immunostaining include Alexa Fluor (AF) (Carter and Shieh, 2015). Immunostaining targets neurons based on particular molecular markers which allows a broad range of targets. Immunostaining is particularly popular as it readily integrates into the modern molecular approaches.

Finally, neurons can be directly injected (Vaney, 2002; Elston, 2003). Direct injection allows neurons to be labeled *in vivo* or in slice samples and later imaged in a fixed preparation, meaning electrophysiology can be related to morphology. It is common to directly inject fluorescent dyes such as Lucifer Yellow (Hanani, 2012) or biotin variants such as biocytin or neurobiotin (Klenowski et al., 2017).

Each method comes with idiosyncrasies and methodological steps that can vary across laboratories. For instance, in immunostaining the antibody concentration, length of incubation time, and accessibility to the antigen all must be balanced to produce a good result (Paavilainen et al., 2010; Carter and Shieh, 2015). All these factors may vary from lab to lab and are a known source of variability. For example, it has been shown that hippocampal CA1 neurons measured in rats housed in different labs are not consistent in terms of their morphometry (Scorcioni et al., 2004). Tripathy et al. (2015) have shown similar biases in electrophysiology (Tripathy et al., 2015; Tebaykin et al., 2017). Understanding the effects of staining is thus crucial for the interpretation of downstream analyses.

Each method also targets different neurons and operates through different biochemical processes such that, even if performed within the same lab, morphology measurements can differ by staining method. For instance, during dehydration it is well-known that incubation with different dyes can affect tissue shrinkage which in turn can affect morphology (Grace and Llinás, 1985). Neurobiotin staining is known to affect both electrophysiology and morphology (Xi and Xu, 1996). In comparing morphology obtained by Golgi-Cox staining and neurobiotin electroporation, it has been shown that neurobiotin-filling revealed significantly larger dendritic arbors and different spine densities compared to GolgiCox-stained neurons (Klenowski et al., 2017). Despite these known issues, there are few systematic studies that examine the size and nature of these biases across the many methods used to quantify morphology.

Large databases of neuron morphologies (Ascoli, 2006) collect data from many labs, each employing different methods. This allows the comparison of data across distinct staining methods. While many experimental aspects of neuron quantification will differ, the staining method is a central experimental choice. As such, it is important to ask what large databases can tell us about the biases induced by staining methods.

Here we quantify the variation in measured neuron morphology related to the staining or the fluorescent labeling method used, though we will refer to both of these as staining method. We analyze rodent data that has been uploaded by various labs to the public morphology repository neuromorpho.org (Ascoli, 2006). We group them based on the biological attributes and the staining methods. By matching on biological attributes and comparing the morphometry of each group we identify the variation that can be explained by different staining methods.

## 2. Methods

### 2.1. Data Acquisition

We used dendrite morphologies submitted to neuromorpho.org (version 7.4), a publicly available database of morphology. We performed a careful search of neuromoropho.org to identify populations of neurons that allow for an appropriate study of the effect of staining method. We describe the search criteria used below.

To ensure that dendrites were traced completely, we filtered out neurons in the database whose *physical integrity* of their dendritic reconstructions was labeled as *incomplete*. We analyzed neurons that are extracted from a healthy animal, by considering only the neurons whose *experimental condition* were labeled as *control*. These steps prevent our analysis from including unwanted effects due to poor reconstruction and experimental condition.

We identified populations of neurons sampled from a specific species, age, gender distribution, region, laminar location obtained with a least two staining methods. To do this we restricted our analysis to the neurons from rat to mouse. We grouped neurons into three age classes: young (more than a month and < 2 months), young adult (between 2 and 6 months) and adult (more than 6 months). To match the gender distribution, we grouped the neurons into three classes: *male, female*, and *male/female*. The latter class was used when labs deposited the equal number of neurons from male and female in the same experiment. We matched the cell types as follows. First, we grouped the neurons into two primary cell types: *principal cell* and *interneurons*. Then we grouped each primary cell types into secondary cell type (including *pyramidal, granule, mitral, GABAergic*). Finally, we grouped them into territory cell type (including *Aspiny, spiny, adult-born, newborn*) if such information was provided. Similarly, we matched the brain regions as follows. We first grouped them by primary brain regions (*neocortex, cerebellum, hippocampus, main olfactory bulb, retina, amygdala, brainstem, entorhinal cortex, spinal cord, protocerebrum*). Then we grouped each primary brain regions into secondary brain regions (including *primary somatosensory, somatosensory, primary visual, CA1, CA, CA3, dentate gyrus, striatum, anterior cingulate, prelimbic, thalamus, hypothalamus, basolateral amygdala*). Then, if each region has a laminar structure (for example somatosensory cortex), we grouped the neurons by their layer [laminar structure contains six distinctive layers (1–6) and three shared layers (2–3, 3–4, 5–6)]. Similarly if the regions had sagittal structure (*left, right*), or coronal structure (*occipital, medial, prefrontal, frontal*) or ventral/dorsal structure then we grouped them accordingly. Brain region definitions and nomenclature are taken from the Allen Institute for Brain Science mouse brain atlas, for both the mouse and rat data (Jones et al., 2009). We use it to normalize neuron assignment at the coarse layer (e.g., *CA1* = subregion of *hippocampus*), as the fine structure of the neuron locations is typically not reported. We omitted neurons for which at least one of the above labels was not reported in the database. Using these criteria we grouped the neurons into classes.

**Figure 1:**
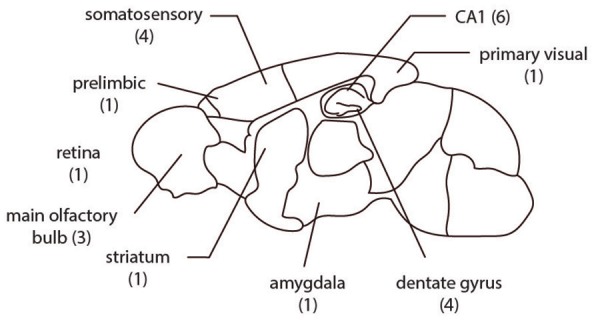
The spatial distribution of the 22 groups analyzed are from multiple brain regions in rodents. The number of comparison groups in each region are listed in parentheses.

If there were at least two different staining methods in a matched group (same brain region, gender distribution, age, species, cell-type) and each staining method has at least five samples in the set, the group was chosen for comparison. In this way we identified 22 matched sets of neurons sourced from more than 60 papers (Figure 1, Tables 1, 2).

**Table 1:** Details of groups used in analysis.

**Index**	**Primary brain region**	**Secondary brain region**	**Tertiary brain region**	**Primary cell type**	**Secondary cell type**	**Tertiary cell type**	**Species**	**Sex**	**Age**	**#samples**
1	N	SS	L 2/3	p	py	-	m	m	a	67
2	N	SSp	L 2/3	p	py	-	r	m/f	y	29
3	N	SSp	L 5	p	py	-	r	m	a	36
4	N	SSp	L 5	p	py	Thick-tufted	r	m	y-a	43
5	N	VIS	L 2/3	p	py	-	m	m/f	a	57
6	HIP	CA1	-	p	py	-	r	m	a	89
7	HIP	CA1	-	p	py	-	r	m	y	24
8	HIP	CA1	-	p	py	-	m	m	y	41
9	HIP	CA1	-	p	py	-	m	m/f	a	60
10	HIP	CA1	-	p	py	-	r	m/f	y	33
11	HIP	CA1	-	p	py	-	m	m/f	y	43
12	HIP	DG	gL	p	gr	-	r	m	y	80
13	HIP	DG	gL	p	gr	adult-born	r	m	a	35
14	HIP	DG	gL	p	gr	-	m	m	a	63
15	HIP	DG	gL	p	gr	new-born	m	m	a	122
16	AMC	BLA	-	p	py	-	r	m	a	102
17	BG	STR	-	p	medium spiny	-	m	m	a	139
18	MOB	mL	-	p	m	-	m	m/f	y-a	20
19	MOB	gL	-	i	g	adult-born	m	f	a	55
20	MOB	gL	-	i	g	-	m	m/f	y-a	21
21	Retina	gL	-	p	g	-	m	m/f	a	402
22	N	PL	L 2/3	p	py	-	r	m	a	74

*For brain regions, N, neocortex; SSp, Primary Somatosensory areas; VIS, Primary visual area; HIP, Hippocampal regions; CA, Ammon's horn; DG, Dentate gyrus; AMC, Amygdalar capsule; BLA, basolateral amygdala; STR, Striatum; PL, prelimbic; BG, basal ganglia; L, layer; gL, ganglion layer; mL, mitral layer. For cell type: p, principal; i, interneuron; py, pyramidal; gr, granule; g, ganglion; m, mitral. For species, m, mice; r, rats. For gender distribution, m, male; f, female; m/f, male/female. For Age, a, adult, y, young; y-a, young-adult*.

**Table 2:** Source references for each comparison group.

**Index**	**References**
1	Benavides-Piccione et al., 2005; Alpar et al., 2006; Cohen et al., 2013
2	Carrel et al., 2015; Hoffmann et al., 2015
3	Kole et al., 2007; Chen et al., 2014
4	Kole et al., 2006; Kole, 2011; Hamada et al., 2016
5	Longordo et al., 2013; Jiang et al., 2015; D'Souza et al., 2016; Vannini et al., 2016
6	Bannister and Larkman, 1995; Carnevale et al., 1997; Megias et al., 2001; Kole et al., 2004; Dougherty et al., 2012; Chen et al., 2014; Malik et al., 2016; Bezchlibnyk et al., 2017
7	Pyapali et al., 1998; Golding et al., 2005; Scorza et al., 2011; Groen et al., 2014
8	Suo et al., 2012; Beguin et al., 2013
9	Druckmann et al., 2014; Lee et al., 2014; Tyan et al., 2014
10	Pyapali et al., 1998; Mulholland et al., 2015
11	Michaelsen et al., 2010; Mendez et al., 2012; Ster et al., 2014; Tripathy et al., 2015; Zhou et al., 2015; Anstotz et al., 2016; Boillot et al., 2016
12	Arisi and Garcia-Cairasco, 2007; Beining et al., 2017
13	Rihn and Claiborne, 1990; Carnevale et al., 1997; Beining et al., 2017
14	Revest et al., 2009; Winkle et al., 2016
15	Carim-Todd et al., 2009; Qin et al., 2014; Platschek et al., 2016
16	Bergstrom et al., 2010; Padival et al., 2013; Henckens et al., 2015
17	Martone et al., 2003; Cazorla et al., 2012; Qin et al., 2014; Nato et al., 2015
18	Fukunaga et al., 2012; Ke et al., 2013
19	Belnoue et al., 2016; Sailor et al., 2016
20	Burton and Urban, 2015; Quast et al., 2017
21	Chen and Chiao, 2014; Sumbul et al., 2014; Krishnaswamy et al., 2015; Poria and Dhingra, 2015
22	Soares-Cunha et al., 2014; Henckens et al., 2015

In these 22 matched groups of neurons are 19 distinct staining methods, labeled by neuromorpho.org. We grouped these into three types: staining with heavy metals, genetic markers, and immunostaining and direct injection. In the heavy metals group: *Golgi*, and *Golgi-Cox*. In the genetic markers group: *green fluorescent protein, red fluorescent protein, enhanced green fluorescent protein*, and *Tag red fluorescent protein*. In the immunostaining group: *immunostaining, horseradish peroxidase, neurobiotin, biocytin, biocytin & betaIV-spectrin, Alexa Fluor 488, Alexa 647-dextrane, Alexa Fluor 594, OGB-1, biotinylated dextran amine, lucifer yellow, green fluorescent protein, Alexa Fluor 488, immunostaining, green fluorescent protein, immunostaining*.

### 2.2. Morphological Features

To compare neuron morphologies we need to quantify them. The morphology of a neuron is described by a set of points each with a coordinate, diameter and index of its parent point. And a set of edges connecting parent points to their children (Stockley et al., 1993). We used six features to measure the effect of staining. Four features are defined in previous publications and are parts of the L-measure (Scorcioni et al., 2008). Two features are unique to this paper. We classed each feature as either global or local.

**Figure 2:**
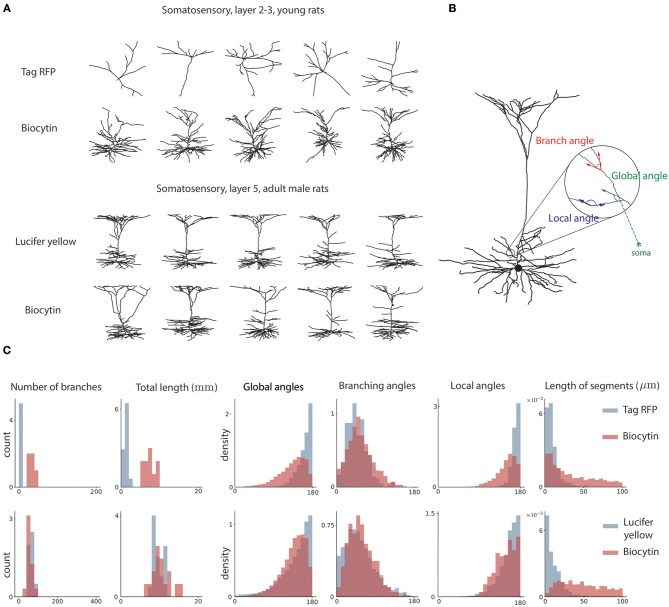
Sample neuron morphologies and features. **(A)** Sample morphologies from two groups of neurons, comparing two distinct staining methods. **(B)** Geometrical features of neuron morphology. Local angle represents the angle between adjacent edges not at a branch point. The global angle represents the angle between an edge and the vector pointing toward the soma. The branch angle represents the angle between two edges that branch from a common node. **(C)** Histograms for the two sample groups of the six morphological features used in analysis.

Three global features are used. First, the number of branching points in the neuron, or how many times the morphology branched. This feature is defined previously in L-measure. Second, the total length of the dendritic tree. This feature is defined previously in L-measure. Third, the global angle. This measures the angle between the dendritic segment and the vector pointing toward the soma. It provides a measure of how much dendrites point away from the soma (Figure 2). This feature is defined in this paper for the first time.

Three local features are used. First, the branching angle. This is the angle between two edges that branch from a common parent. This feature is defined previously in L-measure. Second, the length of segments. This is the length of dendrite between two consecutive branching nodes. This feature is defined previously in L-measure. Third, the local angle. The local angle measures the angles between the vector connecting the node to its parent and the vector connecting the node to its child. We only consider nodes that have one child. This measures how straight the neurites of the neurons are. This feature is defined for the first time.

For features that are measured per dendrite segment (e.g., branching angle, global angle, local angle), data are pooled over all neurons in the group. In order to avoid artifacts due to software reconstruction of the neuron, we resampled the morphology such that the distance between each consecutive node was equal. To do this we preserved the terminals and branching nodes and selected one node every 10 micrometers (but we suppress the last point if it is within 10 micrometers of the terminal or branch node). This way we obtain a normalized representation that can be compared.

### 2.3. Statistical Testing

We tested for an effect of the staining method on each morphological feature, above effects explained through biological attributes. Our morphological features are generally continuous valued, while neuron classes are categorical. Further, the morphological features generally follow a non-Gaussian distribution (Figure 2). This requires using non-parametric tests. We thus used the Wilcoxon rank-sum test. That is, for each group b∈B, we tested:

(1) $$\begin{array}{r} H_{0}\left(B=b\right):\mu_{1/2}\left(M|B=b,S=s_{1}\right)=\mu_{1/2}\left(M|B=b,S=s_{0}\right), \end{array}$$

for all b∈B, where μ_1/2_ represents the median, *S* the staining method, and *M* the morphological feature. The hypothesis that no overall effect exists for a given morphological feature is

(2) $$\begin{array}{r} H_{0}:\cap_{i=1}^{\left|B\right|}H_{0}\left(B=b_{i}\right), \end{array}$$

for the *N* levels in *B*. To correct for multiple testing we used Bonferroni correction.

#### 2.3.1. Average Effects

The differences in morphology between staining methods can also be quantified over groups by considering the difference in means:

(3) $$\begin{array}{l} \beta_{j}=𝔼\left(𝔼\left(M_{j}|B,S=S_{1}\right)-𝔼\left(M_{j}|B,S=S_{0}\right)\right), \end{array}$$

which summarizes the average difference in morphological feature *M*_*j*_. This corresponds to the average treatment effect in the causal inference literature (Pearl, 2009), although we can not (and do not) make claims about causality here. A null distribution for each β_*j*_ is generated by repeated permutation of staining label, allowing us to determine significance levels.

## 3. Results

**Figure 3:**
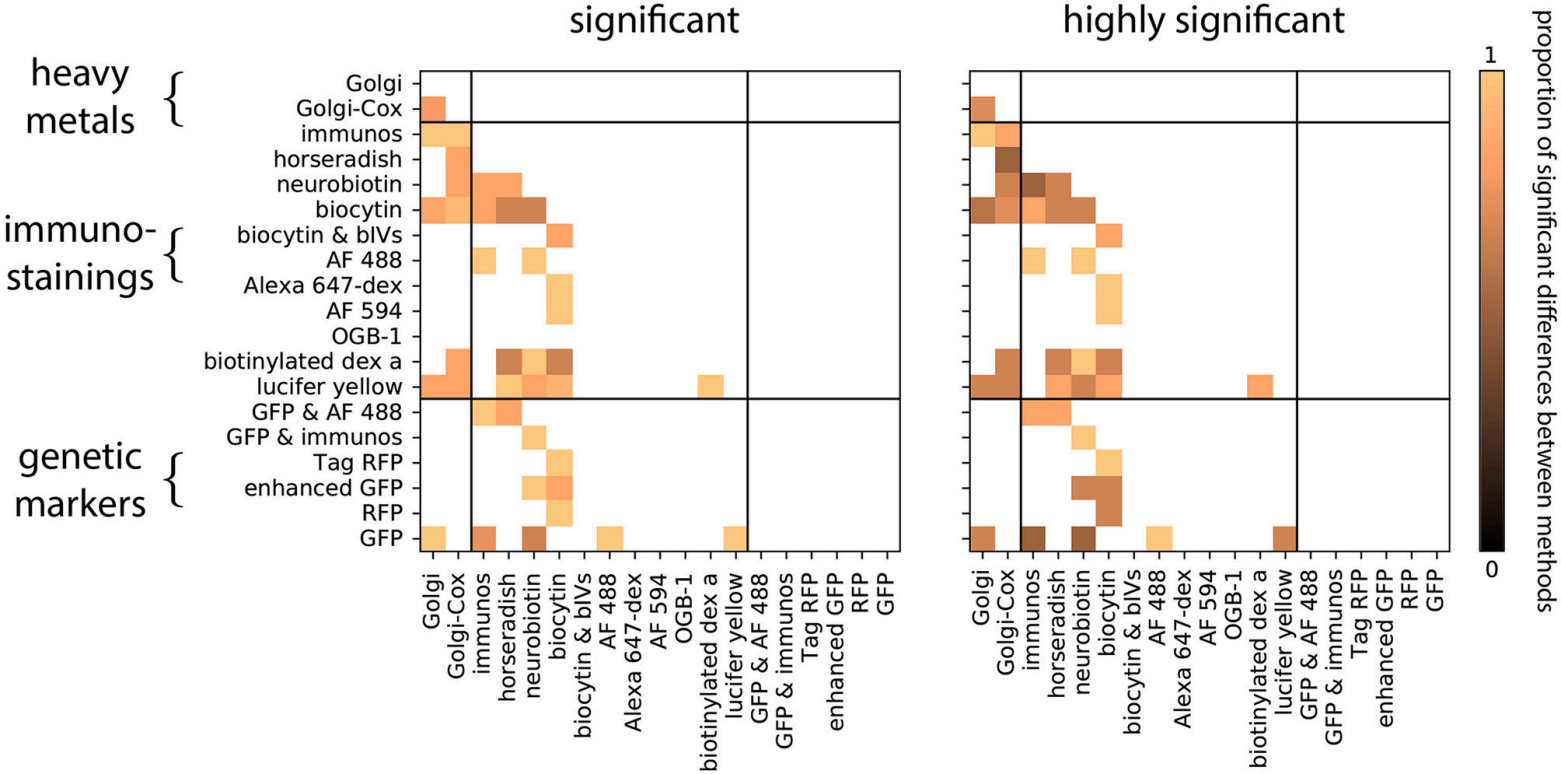
Proportion of significant differences between pairs of staining methods over all groups and morphological features. Zero means there is not any difference between the staining methods among the groups and one means all the groups are different. Computed using the Wilcoxon rank-sum test, corrected (Equation 2). Significant means *p* < 0.05, highly significant means *p* < 0.001.

We first asked if neurons obtained by distinct staining methods are distinguishable. Within each group, we compared the distribution of each morphological feature between a pair of staining methods (Figure 2). To do this we tested the hypothesis that the reconstructed morphologies are statistically similar within each group. We observed that, for each pairwise comparison between two staining methods, there is at least one group which shows significant differences in at least one morphological feature (Figure 3, Wilcoxon rank-sum test, *p* < 0.05, corrected). In fact, for most of the pairwise comparisons between staining methods we observed a large proportion of highly significant differences (Figure 3, Wilcoxon rank-sum test, *p* < 0.001, corrected). This suggests that morphologies obtained by different staining methods seldom agree with each other.

We wondered if the biases in neuronal morphology between staining methods of the same type (e.g., Golgi vs. Golgi-Cox) were less than the biases in morphology between methods of a different type (e.g., Golgi vs. GFP). In fact comparisons between staining methods of the same class showed just as high a proportion of statistically significant differences as comparisons between methods of a different class: in both within-class and between-class comparisons 90% of tests performed were statistically significant (Figure 3). Thus, even morphologies obtained by similar methods can show strong biases due to experimental choices.

**Figure 4:**
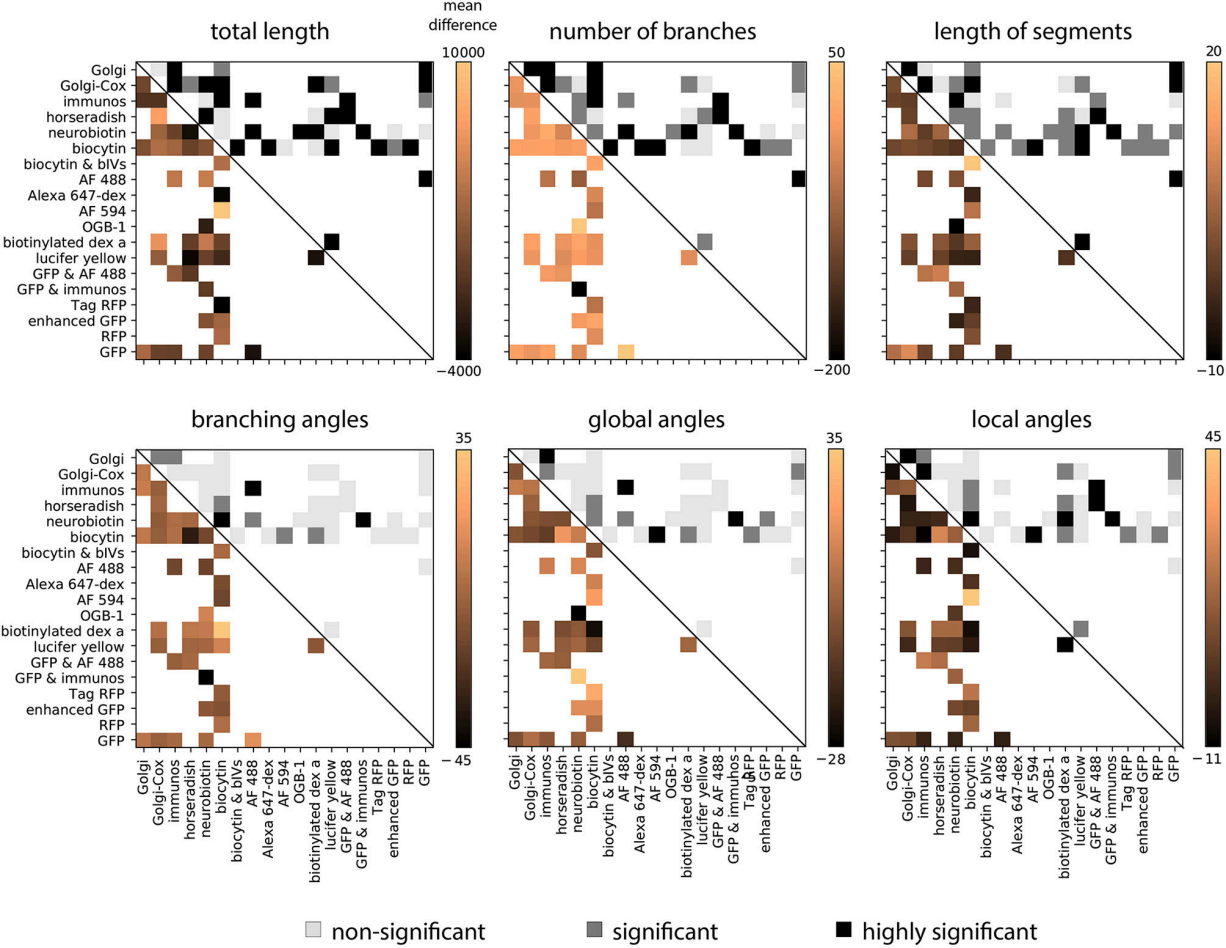
Pairwise average effect sizes for six morphological features. Upper right entries show statistical significance of differences (Equation 3). White squares represent no comparison, light gray squares represent a non-significant difference, gray squares represent a significant difference (*p* < 0.05), and black squares represent a highly significant difference (*p* < 0.001). To compute the significance level the average absolute difference in morphological features is compared with a null-distribution generated through permutation. Lower left entries show average difference in each feature between the two corresponding methods.

Given this preponderance of variability related to the staining method, we sought to understand which morphological features in particular had the strongest biases. In order to examine this we computed the average difference in each feature between each pair of staining methods, averaged over all groups for that comparison. This analysis shows that in general the total length, number of branches, and the length of segments show the strongest biases related to the staining method (Figure 4). Using a permutation test to determine the statistical significance of the average effect, we observed that 76% of average effects within these three features were significant. While features related to angles of the dendritic tree show weaker effects—only 32% of average effects in these features were significant. This suggests that features related to the coverage of the dendritic tree are most affected by the choice of staining method.

An omission from these analyses is the possible confounding effect that the rodent strain may have on neuron morphology (Rodriguez et al., 1999; Routh et al., 2009). There is less available data that we could use for matching. Nonetheless, we also analyzed the effect of the staining method on data matched also by strain. This resulted in six comparison groups. In this analysis the staining method biases are of similar significance and size in comparison to the analysis presented above (Supplementary Figures 1, 2). Thus, the biases we observe in morphology due to the staining method do not appear to be explained by reported differences in neuron morphology between rodent strains.

A caveat of the available data for our analysis is that it cannot fully separate the effect of the staining method from other laboratory-fixed effects. The ideal dataset to uniquely identify the effect of the staining method would be to have one lab perform the same experiments but with different staining methods. There are a few instances in which such a comparison was made. However, while the data we have thus exhibits a threat to causal validity (Pearl, 2009), we do have sufficient data to estimate how many neurons would be required to estimate such an artifact. To do this we performed a power analysis of the Wilcoxon rank-sum test through resampling. For most morphological features, we found that an effect size of *d* = 0.5 required 50 neurons/data points to detect the effect with probability 0.9 (1−β), assuming a type I error rate (α) of 0.05. For features that are defined for each dendrite segment, a single neuron would generally provide this much data. However, to ensure neuron-neuron variability is taken into account, a safer estimate is to assume at least 50 neurons are required. Thus, it seems likely that future versions of neuromorpho.org will soon be able to answer these questions with more precision.

## 4. Discussion

It is important to accurately characterize neuron morphology for a number of reasons. Dendritic morphology determines the computations a neuron can perform, and has a role in circuit function and neurological disease (Agmon-Snir et al., 1998; Elston and Fujita, 2014; Šišková et al., 2014; Yang et al., 2017). Morphology varies by brain region, cell layer, species, and age (Scheibel and Jacobs, 2003; Elston, 2007; Spruston, 2008; Elston and Fujita, 2014). It thus may provide clues as to the function of the region. For instance, Purkinje cells in the cerebellum and pyramidal cells in cortex may provide striking examples of a structure-function relation (Stein and Glickstein, 1992; Körding and König, 2001; Guerguiev et al., 2017). And distinct morphological features affect functional properties differently. For instance, features to do with dendrite diameter may affect electrophysiological properties more than branching angle. Characterizing the morphology specific to brain region, species, etc. is thus important. This is most cleanly identified when the same experimental methodology is used over different brain regions. For instance, Jacobs and Scheibel studied dendritic variation in primate cortical pyramidal cells with the Golgi technique (Scheibel and Jacobs, 2003). Elston and colleagues studied thousands of individually injected cells from multiple cortical areas in a singe hemisphere, replicating the studies in age/sex/hemisphere matched brains within a species and across species (Elston et al., 2001; Elston, 2007). Yet many brain regions and cell types have not been analyzed in this form. Studying variability in morphology by brain region and cell type must typically be performed with data collected from many different methods. Before conclusions can be made, the bias of the methodology must therefore be established.

Here we focused on the correlation of staining methods on measured neuron morphology. We showed a significant difference between neurons that were extracted from the same region, species, gender distribution, and age but with different staining methods. Although this analysis was focused on the staining method, a similar approach could be taken to study the effect of other methodological details such as the reconstruction software or microscopy method. Understanding the source of these artifacts is necessary for us to have an accurate picture of the variation of neurons in the brain.

There are a number of explanations for biases in morphology related to staining methods. First, there may be procedural differences between laboratories, coming from preferences for particular sub-regions or cell types or other preparation details not reported. Indeed previous studies show this is a large source of variability (Scorcioni et al., 2004). Large differences in morphology can exist within a small region, e.g., visuotopic variation within visual cortex and age (Elston, 2003, 2007; Elston and Fujita, 2014). As such, we may expect significant biases to be related to non-staining related signals.

Second, there are methodological biases related to the 3D or planar reconstruction of neuron morphology. To produce a 3D reconstruction of a neuron, we need to fix a direction for the slicing the specimen and choose the thickness of the slices. The staining methods may set a limit on the slice thickness. For instance, when using GFP, neurons are often imaged through confocal microscopy. This sets a bound for the slice thickness, which may affect morphometrics (Rodriguez et al., 2003; Ke et al., 2013). Shrinkage of the neuron during the fixation can also affect measured morphology (Grace and Llinás, 1985). These biases most likely affect the local morphological features, such as angles; the global features, such as total length, are likely less affected. Yet we observed larger biases in global features. This may suggest that biases related to 3D reconstruction are minimal.

Third, there may exist differences caused by other methodological details that happen to be correlated with the staining method, not because the method goes in hand with the staining method itself, but just by chance (or cultural heritage) in the data we analyzed. For example if the objective type used in the microscopy correlates with different staining methods then that would be a potential confound. However, by performing the same comparison over lab groups and brain regions, we mitigate these confounding effects to some extent, and thus better measure differences that are particular to the staining method. But these other explanations can not be ruled out entirely without more controlled comparisons. This is challenging, even with a large database such as neuromorpho.org. Our power analysis demonstrates how much data would be needed to cleanly address these questions. Ultimately, only a clean experiment with a proper random assignment strategy could produce causal certainty.

Our results are consistent with one mechanistic account by which biases are created from the staining method, and not other potential confounds: different molecule size of agents used in different staining methods target different parts of a neuron. If different staining methods capture different parts of the morphology then we would expect strongest artifacts to be observed between features to do with the amount of dendritic tree described, e.g., total length, dimension, number of branches, etc, and smaller artifacts for more local geometric properties like the branching angles. If, alternatively, the biases observed in morphology were due to staining methods/labs targeting different neuronal subpopulations within a given brain region, then we may expect stronger artifacts to also be observed in local parameters such as branching angle. We do not observe strong artifacts in these parameters. A more careful modeling approach that takes generated neuron morphologies and subsamples them according to a staining model may be able to give a more precise account of the type of biases we may expect due to the staining method, and thus this interpretation could be better tested.

The feature set used here is often used as a basis of cell classification (Vasques et al., 2016). In this regard, our results suggest the need to standardize and carefully characterize these artifacts–after all, such biases could have massive effects on the results of clustering methods used for cell type identification. Alternatively, although some features are affected by different staining method, there are some features that are only weakly affected by the method (Figure 4). It would thus be possible to use features that vary most by cell type and least by staining method as the basis of classification or clustering. This should allow combining our findings with those of previous classification approaches to make the procedures robust to the details of the staining method.

Our analysis tells a cautionary tale about the progressively more popular combination of data sets across labs. Fully characterizing neuronal morphology and its relation to function relies on the generation and analysis of vast amounts of data. Across neuroscience, collaborative efforts across institutions are studying morphology (e.g., Churchland, 2017). Amongst the wealth of datasets available, the need for understanding variability due to the data generation process is important for drawing inferences and analyzing data across disparate sources. This problem is becoming widespread in neuroscience where electrophysiological, molecular, and morphological data are now routinely shared.

## Data Availability

The datasets analyzed for this study can be found in repository neuromorpho (version 7.4) http://neuromorpho.org. The code for performing the analysis is available at https://github.com/BonsaiNet/Staining-methods-and-morphologies.

## Author Contributions

RF and KK designed the study. RF and BL performed the analysis. RF, BL, and KK wrote the manuscript.

### Conflict of Interest Statement

The authors declare that the research was conducted in the absence of any commercial or financial relationships that could be construed as a potential conflict of interest.
