# Supplementary material for: Quantifying How Staining Methods Bias Measurements of Neuron Morphologies
Source: Front Neuroinform. 2019 May 21;13:36. doi: 10.3389/fninf.2019.00036 (PMC6541099; doi:10.3389/fninf.2019.00036)
Supplement: Supplementary file 1 [file Image_1.pdf]

1 Supplementary Material

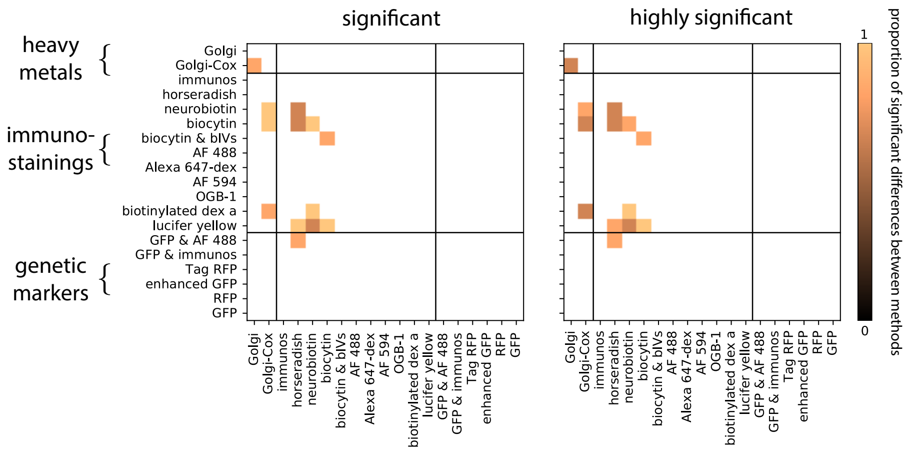

Figure 1: **Proportion of significant differences between pairs of staining methods over all groups and morphological features.** The groups are obtained with the same procedure as explained in the method section, with an extra matching on the strain. We restrict the analysis to four different strains (*C57BL/6*, *Sprague-Dawley*, *Wistar*, *C57Bl6/129SvEv*). This results in six groups. In the figure, zero means there is not any difference between the staining methods among the groups and one means all the groups are different. Computed using the Wilcoxon rank-sum test, corrected (Eq. (2)). Significant means $p < 0.05$, highly significant means $p < 0.001$.

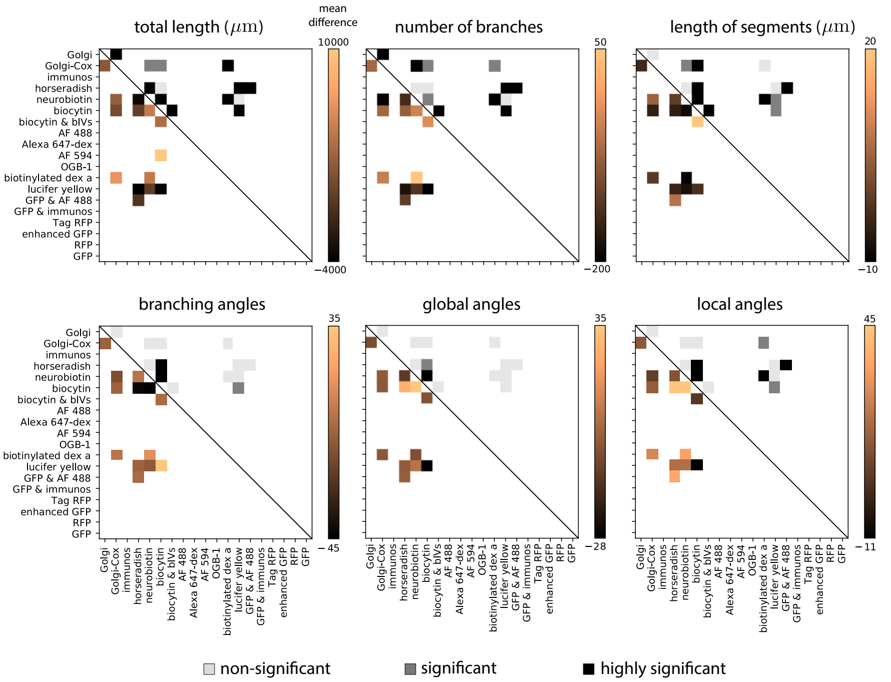

Figure 2: **Pairwise average effect sizes for six morphological features.** Upper right entries show statistical significance of differences (Eq. (3)). The groups are obtained with the same procedure as explained in the method section, with an extra matching on the strain. We restrict the analysis to four different strains (*C57BL/6*, *Sprague-Dawley*, *Wistar*, *C57Bl6/129SvEv*). White squares represent no comparison, light gray squares represent a non-significant difference, gray squares represent a significant difference ($p < 0.05$), and black squares represent a highly significant difference ($p < 0.001$). To compute the significance level the average absolute difference in morphological features is compared with a null-distribution generated through permutation. Lower left entries show average difference in each feature between the two corresponding methods.
